# Barriers to voluntary participation in sport for children: a systematic review

**DOI:** 10.1186/s12887-018-1014-1

**Published:** 2018-02-09

**Authors:** Sarah Somerset, Derek J. Hoare

**Affiliations:** 1 National Institute for Health Research (NIHR) Nottingham Biomedical Research Centre, Ropewalk House, 113 The Ropewalk, Nottingham, NG1 5DU England; 2 Otology and Hearing Group, Division of Clinical Neuroscience, School of Medicine, University of Nottingham, Nottingham, NG7 2UH England

**Keywords:** Sport, Participation, Barriers

## Abstract

**Background:**

Numerous studies have detailed the physical health benefits of children’s participation in sport and a growing body of research also highlights the benefits for mental health. Children who participate in sport have also been shown to be advantaged academically. However, despite the benefits there is evidence that children are leading increasingly sedentary lifestyles and are at greater risk of chronic disease than those with active lifestyles. Sport provides an important means for children to achieve their recommended amount of daily physical activity. This systematic review asks ‘what are those barriers to children’s participation in sport?’

**Methods:**

Literature searches were carried out in June 2015 using; EMBASE, Medline, CINAHL and SportDiscus using the search terms barrier*, stop*, prevent*, participat*, taking part, Sports/, sport*, “physical education”, PE, child*, young person*, adolescen*. These were supplemented with hand searches. A total of 3434 records were identified of which 22 were suitable for inclusion in the review, two additional studies were identified from Google Scholar in November 2016. Both qualitative and quantitative studies were included. Study’s included in the review assessed children up to 18 years of age. Study quality was assessed using Critical Appraisal Skills Programme (CASP) tools.

**Results:**

Studies took place in the school environment (*n* = 14), sports club (*n* = 1), community setting (*n* = 8) and adolescent care setting (*n* = 1). Frequently reported barriers across quantitative studies were ‘time’ (*n* = 4), ‘cost’ (*n* = 3), ‘opportunity/accessibility’ (*n* = 3) and ‘friends’ (*n* = 2). Frequently reported barriers across qualitative studies were ‘time’ (*n* = 6), 'cost' (*n* = 5), 'not being good at sport' (*n* = 6) and ‘fear of being judged/embarrassed’ (*n* = 6).

**Conclusion:**

Policy makers, parents and teachers should all be aware that ‘cost’ and ‘time’ are key barriers to participation in sport. More local sports opportunities are needed where costs are reduced. Schools and local clubs could better work together to provide more affordable local opportunities to increase children’s participation in sport.

**Electronic supplementary material:**

The online version of this article (10.1186/s12887-018-1014-1) contains supplementary material, which is available to authorized users.

## Background

Sport is defined as an *“an activity involving physical exertion and skill in which an individual or team competes against another or others for entertainment”* [1]. Sport can involve moderate or vigorous physical activity. Sports involving moderate physical activity include those such as badminton or cricket, where a person can converse easily at the start of play but breathing becomes more effortful as they continue play. Sport involving vigorous physical activity includes those such as competitive swimming where there is exertion and physical demands are high, e.g. on the person breathing [1]. For most children physical education (PE) provides the first exposure to sport [2, 3] and it is likely that this early exposure is very influential of their participation in later years [4].

Children show benefit from participation in sport in terms of mental and physical health and school performance [5–8]. Numerous studies detail the physical health benefits of participation in sport and there is also a growing body of research investigating the psychological and mental health benefits [9, 10]. Children who participate in sport are shown to score higher on scales for happiness, mental health and physical health compared to those not participating in sport [7]. Regular participation in sport has also been linked to better quality of life [9]. However, despite all the known benefits, children are also leading increasingly sedentary lifestyles, associated with increased risk of obesity and chronic diseases such as diabetes and coronary artery disease [11, 12].

Sports are an important means for children and young adults to gain their recommended level of physical activity [13]. The Health and Safety Executive (2012) state vigorous activities (those strengthening muscle and bone such as swimming, running or football) should be carried out on at least three days per week [13]. The WHO [14] guidelines for physical activity for children and young people aged 5 to 17 years is for at least 60 min of exercise such as swimming, tennis, rugby, football or squash per day. [13, 15, 16]. Boys participate in sport more frequently than girls and are more physically active from childhood into adolescence [13, 17–19]. A study in Europe focusing on children aged 9 to 15 years showed sports participation decreased across all ages in all countries [20]. Worryingly this pattern is seen across the world with global estimates showing that 80% of 13 to 15 year olds do not meet the guided amount of physical activity including sport [21].

For the purposes of this review physical education (PE) is also considered part of sport. This review does not focus on physical activity but instead views sport as a subset of physical activity.

Previous research in the UK found that from ages ~ 4–17 years 55% of children took part in at least 3 h of PE and out of hours school sport, but this decreased when children moved from primary (ages ~ 4–12 years) to secondary (ages ~ 12–18 years) school in the UK [22]. Quick et al. [22] conducted a series of surveys to establish the proportion of pupils receiving two hours of curriculum PE and the proportion of pupils participating in at least three hours of high quality sport and PE in a normal week [22]. In broad ‘physical activity’ terms, barriers such as ‘preferences and priorities’, ‘family life’ and ‘parental support’ can influence levels of sports participation [23]. Allender et al. [4] identified ‘being highly structured’ and ‘being a competitive actvity’ as potential barriers to participation in sport and other physical activity in young children.

In terms of facilitators of sports participation much of the current literature focuses on more specific barriers such as those faced by people with physical disability, visual impairment, or those in economically disadvantaged areas [24–26]. There are few current studies which examine facilitators in the more general population [27]. This may be a reflection in part that the evidence for what general barriers children face when they wish to participate in sports has yet to be synthesised. Here we systematically review studies primarily concerned with identifying general barriers to voluntary sports participation faced by all children and consider how these barriers might best be addressed.

The aim of this systematic review was to identify and synthesise the primary evidence on barriers to voluntary sports participation that are faced by children, and to then consider how those barriers might best be addressed.

## Methods

The protocol for this systematic review was prospectively registered on PROSPERO (CRD42015023993) and is reported according to the PRISMA guideline (http://www.crd.york.ac.uk/PROSPERO/display_record.asp?ID=CRD42015023993).

### Searches

Literature searches were carried out in June 2015 using four electronic databases; EMBASE, Medline, CINAHL and SportDiscus using the search terms barrier*, stop*, prevent*, participat*, taking part, Sports/, sport*, “physical education”, PE, child*, young person*, adolescen*. An example search can be found in Appendix. Authors of the systematic reviews identified in the initial searches were also contacted to see if they were aware of any other relevant studies. Searches were updated in November 2016 with an additional search of Google Scholar. Search terms were simplified for Google Scholar to child*, barrier*, sport, and participation [28, 29]. A stopping rule was prospectively applied to the Google Scholar search results whereby screening of titles and abstracts was stopped after three consecutive pages where no new records were taken forward to full text screening.

### Inclusion / exclusion

This review is specifically focused on the barriers to children’s voluntary participation in sport. Only peer-reviewed records, describing original research and available in English, were included. Studies were required to discuss barriers to voluntary participation in sport in children up to the age of 18 years.

Studies were excluded if they only concerned the impact of non-participation in sport or the effects sports participation can have on variables such as the female athlete triad, smoking, or alcohol consumption. Studies where sport was included as an intervention (i.e. ‘forced’ participation) were excluded. Studies were also excluded if they only reported on participants with additional needs or were focused on injury from sports participation or pre exercise testing. Guidelines, policy documents, and other non-peer-reviewed publications were excluded. Studies were excluded if they did not investigate barriers to participation in sport or were not about participation in sport. Both qualitative and quantitative records were included.

### Study selection

A title screen was carried out by researcher 1 (SS) to remove any duplicates from the searches. Abstract screening was conducted independently by both authors. Any studies identified by either researcher as either providing likely or unclear evidence for inclusion were retrieved for full text review. The full texts were then independently reviewed against the inclusion/exclusion criteria by both authors. A consensus meeting was held to determine the extent of agreement and to resolve any disagreement, and agree the records to be included.

### Data extraction

A data extraction form was developed, piloted on four records, and revised before data extraction began. Both author’s independently extracted data, and discrepancies were reviewed and resolved through discussion and revisiting the record. Extracted data included author, year of publication, country or location of study, study design, number of participants, age range of children in the study, type of barrier to participation, socioeconomic information, type of sport, and whether sport took place in or out of school.

### Study appraisal

To appraise quantitative studies we used the Clinical Appraisal Skills Programme (CASP) tool for cohort studies [30]. This tool contains 12 questions. Questions 3, 4, 6 and 12 were not used however as they are only relevant to intervention studies (Table 1). For each question there are three response ratings: ‘yes’, ‘no’, or ‘can’t tell’.

**Table 1:** CASP appraisal for quantitative studies

		Boiche 2009 [46]	Casper 2011 [47]	Dollman 2010 [48]	Gordon 1996 [32]	Gracia-Marco 2010 [70]	Hardy 2010 [35]	Irwin 2009 [34]	Kirshnit 1989 [33]	Perry 2013 [49]
1	Did the study address a clearly focused issue?	Y	Y	Y	CT	Y	Y	Y	Y	Y
2	Was the cohort recruited in an acceptable way?	Y	Y	Y	Y	Y	Y	Y	Y	Y
5a	Have the authors identified all important confounding factors?	N	Y	Y	N	N	N	N	N	N
b	Have they taken account of the confounding factors in the design and / or analysis?	N	Y	Y	N	N	N	N	N	N
8	How precise are the results?	Y	Y	Y	N	Y	Y	N	N	Y
9	Do you believe the results?	Y	Y	Y	Y	Y	CT	Y	Y	Y
10	Can the results be applied to the local population?	Y	Y	Y	N	Y	N	N	N	Y
11	Do the results of this study fit with other available evidence?	Y	Y	Y	N	Y	N	Y	Y	Y
7	What are the results of this study?	Satisfaction and value with sport are predictors of dropout	Time viewed as biggest participation barrier to sport	Girls from poorer background experience a lack of parental support	Barriers to sport participation in leisure time are cost and opportunity	Males engaged in more extracurricular activity than emales	Cost and time influence parents decision for their child’s participation in sport	Income has an impact on swimming ability (due to lessons taken)	Younger boys spend more time in sports than older boys. Girl’s time did not differ. Results inconsistent	Participation in after school sport associated with satisfying the recommended daily PA

*Y* Criteria was met, *N* Criteria not met, *CT* Cannot tell if criteria was me

For qualitative studies we used the Clinical Appraisal Skills Programme (CASP) tool for qualitative studies [31]. This tool contains ten questions (Table 2). For each question there are three response ratings: ‘yes’, ‘no’, or ‘can’t tell’.

**Table 2:** CASP appraisal for qualitative studies

		Armentrout 2011 [51]	Azzarito 2012 [37]	Barnett 2013 [38]	Basterfield 2016 [54]	Dismore 2010 [36]	Eime 2010 [39]	Eimear Enright 2010 [40]	Fisette 2013 [41]	Holt 2011 [42]	Kimm 2006 [52]	Oliver 2009 [43]	Quarmby 2011 [55]	Stanely 2012 [44]	Totaro-Garcia 2011 [45]	Wetton 2013 [53]
1	Was there a clear statement of the aims of the research?	Y	N	Y	Y	Y	Y	Y	Y	Y	Y	Y	Y	Y	Y	Y
2	Is a qualitative methodology appropriate?	Y	Y	Y	Y	Y	Y	Y	Y	Y	Y	Y	Y	Y	Y	Y
3	Was the research design appropriate to address aims of research?	Y	Y	CT	Y	CT	Y	Y	Y	Y	Y	Y	Y	Y	Y	Y
4	Was the recruitment strategy appropriate?	Y	CT	Y	Y	CT	Y	Y	Y	Y	Y	Y	Y	Y	Y	Y
5	Was the data collected in a way that addressed the research issue?	Y	Y	CT	Y	Y	Y	Y	Y	Y	Y	Y	Y	Y	N	Y
6	Has the relationship between researchers and participants been adequately considered?	N	Y	N	Y	CT	N	N	CT	N	N	Y	N	N	N	N
7	Have ethical issues been taken into consideration?	N	Y	Y	Y	Y	Y	Y	Y	Y	Y	Y	Y	Y	Y	N
8	Was the data analysis sufficiently rigorous?	Y	CT	Y	CT	CT	Y	Y	Y	Y	Y	Y	Y	Y	N	N
9	Is there a clear statement of findings?	Y	Y	Y	Y	Y	Y	Y	Y	Y	Y	Y	Y	Y	Y	Y
10	How valuable is this research?	Y	N	CT	CT	CT	Y	CT	CT	Y	CT	CT	Y	N	CT	Y

*Y* Criteria was met, *N* Criteria not met, *CT* Cannot tell if criteria was met

Study appraisal was conducted independently by the two authors and any disagreements in scoring were resolved through discussion and revisiting the record.

## Results

**Fig. 1:**
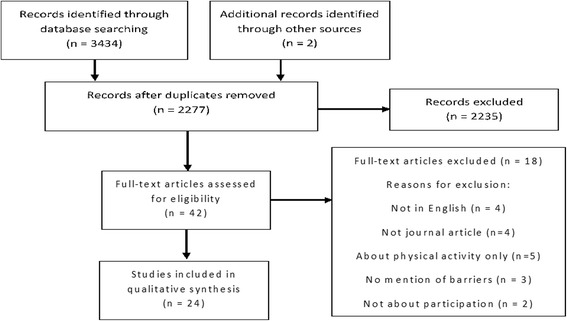
PRISMA flow diagram PRISMA flow diagram showing the records identified, duplicates removed, full text articles assessed for eligibility and studies included

The initial searches produced 3434 records of which 22 studies met the criteria for inclusion; 9 quantitative studies and 13 qualitative (*see* Fig. 1 for flow diagram). An additional search of Google Scholar in November 2016 identified two additional studies which met the criteria for inclusion, one qualitative and one mixed methods. Study characteristics are given in Tables 3 and 4. Studies took place in the school environment (*n* = 14), sports club (*n* = 1), community setting (*n* = 8) and adolescent care setting (n = 1). Of the studies nine quantitative and eight qualitative studies made use of male and female participants whilst seven of the qualitative studies had female only participants. Socioeconomic information was reported in five of the quantitative studies and nine of the qualitative studies.

**Table 3:** Study characteristics for the quantitative studies included in the review

Author & Year	Research Aim	Method	Sample	Country	Age (Years) or School Grade	Sport	Socioeconomic info	Barriers Identified	Negative causal/association relationship
Boiche 2009 [71]	To examine simultaneously several potential determinants of dropout in sport or continuation of sport	Questionnaire (tested athelets views of their experience of sport, coaches, team mates and parents)	261,228 (62%) M 139 (38%) F	France	Mean age 14.6	General Outside school	NR	Personal goal conflict Lack of parental support Clash of or inapproprisate prractice times Location too far to travel Perceived competence – child feeling they are no good at sport Bad or negative relationship with coach	Causal
Casper 2011 [47]	To establish the most (and least) important reported constraints overall, to establish the relationship between perceived constraints and prior sports participation and identify how perceived constraints differ across sociodemographic groups	Questionnaire (made use of a 25 items scale assessing 3 theoretical dimensions, intrapersonal, interpersonal and structural constraints)	2465 1169 (50%) M 1169 (50%) F	USA	6th, 7th, 8th graders	General	52% caucasian (1103), 36% African American (747), 11% Latino (237). 32% (739) received free or reduced price lunch. 1591 no reduced lunch	Barriers predefined: Lack of time No one to partner with Lack of facilities Lack of accessibility	Causal
Dollman 2010 [48]	To identify relationship between socioeconomic position (SEP) and sport participation and to explore SEP gradients in personal, social and environmental mediators of participation	Cross-sectional Survey (Children listed participation in organised sports over the previous 12 months, the season they participated in and the context. The Children’s Physical Activity Correlates (CPAC) Scale was administered.)	3434 786 (45%) M 951 (55%) F	South Australia	Grades 5–10	General	51% metropolitan schools, 49% rural schools. High socioeconomic background: M 36.6%, F 35.1%. Medium socioeconomic background: M 27%, F 29.3%. Low socioeconomic background: M 36.4%, F 35.5%	Barriers predefined: Level of parental support for females Low socioeconomic background for females placed restriction on sport participation. Boys from low socioeconomic background reported less access to organised sport	Association
Gordon 1996 [32]	To examine the leisure activity involvement, deviant activity, reasons for participation, suggestions for leisure service provision, leisure satisfaction, and perceived control/barriers to leisure fur adolescents from two Queensland areas, one an urban city and the other a small rural region.	Survey (to gain insight into youth’s leisure time use, activity choices, reasons for involvement in activity, situational factors, satisfaction, dissatisfaction with leisure time use)	140 students	Australia	Grades 8, 10, 12	General Outside School	NR	Lack of support from parents / guardians Lack of opportunities, Lack of transport, Cost too high Laziness, Lack of friends	Association
Gracia-Marco 2010 [70]	To highlight the sports that are practised most as extra-curricular activities, identify differences between the sexes in extra-curricular participation in sports, and determine its association with body fat and socio-demographic factors.	Survey (to determine extra-curricular participation in sports, also made use of 2 anthropometrics in each city measuring, weight, height, skinfolds and circumferences)	2165 Students 1124 (52%) M 1041 (48%) F	Spain	13–18	General Outside School	NR	Being female rather than male	Association
Hardy 2010 [35]	To explore parents’ perceptions of factors related to time, costs and activity choice which may influence their decision to allow their child to participate in organised sports, and to describe parent’s recent expenditure on children’s sport-related items.	Questionnaire (Asked parents if their child had participated in sport in the last month, if factors such as cost, travel and availability would make them more or less likely to allow their child to participate in organised sports activity and whether they had paid for any sports-related expenditure in the past 3 months)	402 (parents) 22% Fathers 78% Mothers	Australia	5–17	General Outside School	Half of households had income less than $80,000 Australian	Location too far Lack of time Convenience to parents Lack of availability Cost too high	Association
Irwin 2009 [34]	To determine variables influencing swimming participation among underrepresented youth.	Survey (drawn from physical activity constraint studies was designed in collaboration with the study’s sponsor, USA Swimming and reviewed by expert panel)	1680, 848 (50.5%) M 832 (49.5%) F	USA	4–17	Swimming Outside school	NR	Not feeling safe Fear of drowning Lack of encouragement from family	Causal
Kirshnit 1989 [33]	To determine sports participation meanings and experiential outcomes according to context, age and gender.	Survey (used experience sampling method and participants carried a pager for 1 week, in response to a pager signal they filled out a self-report form)	401	USA, Chicago	5th to 9th Grade	General In and out school	Working and middle class communities	Lack of choice	Association
Perry 2011 [49]	To identify intrapersonal perceptions of motivators and barriers to PA, behavioural factors and environmental factors of opportunities for PA that are associated with meeting recommended levels of PA.	Survey (Included questions about use of 13 parks and schoolyard in the local area, questions relating to risk behaviours and protective factors and the rest of the survey questions were drawn from the Washington State Healthy Youth Survey)	773,379 (49%) M 394 (51%) F	USA	Grades 6, 8, 10, 12	General	30% below federal poverty level, 100% on free or reduced cost lunch program	Lack of time Lack of transport	Causal

*NR* Not reported, *M* Male, *F* Female, *PE* Physical education, *PA* Physical activity

**Table 4:** Study characteristics for the qualitative studies included in the review

Author & Year	Research Aim	Method	Sample	Country	Age (Years) or School Grade	Sport	Socioeconomic info	Barriers Identified	Negative causaul/association relationship
Armentrout 2011 [51]	To establish a clear and specific understanding of organisational barriers and personal reasons that may lead youth to discontinue sport participation and to determine changes that could be made to lead to continued involvement.	Survey open ended questions	237 parents/guardians of children who had been youth hockey players	USA Minnesota	4–17	Ice Hockey Outside school	NR	Lack of time Cost too high Location too far Availability of ice rink Politics affecting participation Lack of enjoyment Lack of interest	Causal
Azzarito 2013 [37]	To explore the geographical dimensions of ethnic-minority girls‟ moving bodies as manifested in relevant spaces and places of their daily lives	Visual ethnography with 2 interviews	20 females	United Kingdom Midlands	14–15	PE Inside School	19 ethnic minority F, 1 white F	Fear of humiliation Self-consciousness Competitiveness Negative appraisal Conformity	Association
Barnett 2013 [38]	To explore adolescents’ perception of the relationship between movement skills, PA and sport, and whether their perceptions differed according to extent of participation in organised PA.	Focus groups	33 17 (52%) M 16 (48%) F	Australia	16–18	General Outside school	99% below average Australian socioeconomic status	Not being good at sport Cost too high Lack of time No Encouragement Lack of resources Fear of being judged	Causal
Basterfield 2016 [54]	To investigate how perceived barriers to participation in school and outside school sports club change in the same cohort over 3 years. Three main hypothesis were tested: 1. Perceived barriers will change from 9 to 12 years, 2. Overweight children will perceive different barriers to children of healthy weight, 3.girls will perceive different barriers than boys	Survey with open ended questions	441,210 (48%) M 231 (52%)	England	9 and 12 years	General	Socioeconomically representative of Northern England	Cost too high Distance to training Lack of facility Lack of time Being shy Doesn’t like being a teacher Doesn’t like strangers Being bullied Lack of skill Fear of getting hurt Fear of making a mistake	Causal
Dismore 2010 [36]	To investigate children’s attitudes toward PE and school sport?,	Interview	10 5 M 5 F	United Kingdom	Year 7	PE Inside School	Mixed state and grammar schools	Conforming to social groups Lack of access to (good) equipment School PE curriculum	Causal
Eimear Enright 2010 [40]	To investigate what a negotiated PE curriculum process looks like, and how students’ increased involvement in curricular decision-making impacts on their engagement with physical education	Participatory action research	41 F	Ireland	14–19	PE	NR	Lack of voice and choice	Causal
Eime 2010 [39]	To use the socioecological model to investigate the broad range of factors which individually and interactively affect participation in sport and PA for currently active rural girls.	Focus groups	27 F	Australia	16 to 17	General	Socio-Economic Indexes for Areas index scores 913–1034	Lack of enjoyment Lack of time Lack of confidence Self-conscious Lack of motor skills Willingness of parents to travel Limited community support Lack of opportunity Limited sporting ability Distance	Causal
Fisette 2013 [41]	To explore girls’ self-identified barriers to theirengagement in and enjoyment of PE.	Focus groups and interviews	7 F	USA	14–15	General	Middle class	Boys dominating sport Conforming to gender stereotype Risk of embarrassment	Causal
Holt 2011 [42]	To examine low-income parents’ and their children’s perceptions of the benefits associated with participation in youth sport.	Interviews	17 parents, 18 children 2 Fathers, 15 Mothers 11 (61%) M 7 (39%) F	Canada	Mean age 12.5	General Outside school	Lowest socioeconomic status bracket in receipt of specific funding to support child’s participation in sports	Cost (in addition to training) Lack of time Transport	Association
Kimm 2006 [52]	To identify barriers to activity participation during adolescence in a biracial cohort of sedentary girls	Questionnaire	2379 F	USA	9–19	General	NR	Lack of time Tiredness No one to go with Embarrassment May get hurt Medical condition Being bad at sport	Causal
Oliver 2009 [43]	To understand 5th-grade girls’ self-identified barriers to physical activity and ways of negotiating those barriers	Feminist active research	11 F	USA	10–11	General Inside school	96% and 81% in each school were economically disadvantaged	Conforming to gender stereotype Boys domination of sport space Boys attitudes to girls in sport	Causal
Quarmby 2011 [55]	To explore psychosocial and environmental factors that contributed to children’s participation in physical and sedentary activities.	Survey & Semi structured interviews	381 (30 from this participated in interviews)	United Kingdom Midlands	11–14	General	NR	Family (single parents, step parents, married parents etc.)	Causal
Stanley 2012 [44]	To explore children’s perceptions of the factors influencing their engagement in PA during the lunchtime period,	Focus groups	54 23 (43%) M 31 (57%) F	South Australia	10–13	General Inside school	Range purposefully sampled, 20% low socioeconomic background	Access to space Perceived competence Suitability of space Lack of time Weather Cost too high Dislike of uniform	Causal
Totaro Garcia 2011 [45]	To identify the physical activity characteristics of adolescents attending thePhysical Education service of CAAA, Department of Pediatrics, Universidade Federal de São Paulo,	Interview Data	118 51 (43%) M 67 (57%) F	Brazil	10–19	General Inside school	NR	Lack of support (situational) Personal Lack of resources (finance and material)	Causal
Wetton 2013 [53]	To gain a greater understanding of these issues which may help, in the future, to develop interventions to increasing team sports participation in girls.	Survey / semi-structured interview	60 F	United Kingdom Midlands	15–16	General Inside and outside school	NR	Conforming to stereotypes Time Bad experience in PE Teacher not supportive Lack of ability Peer disapproval Other hobbies Gender Stereotype	Causal

*NR* Not reported, *M* Male, *F* Female, *PE* Physical education

The quantitative studies included took place in France (*n* = 1), Australia (*n* = 3), USA (*n* = 4) and Spain (*n* = 1). The qualitative studies included were conducted in Australia (*n* = 3), Brazil (*n* = 1), Canada (*n* = 1), Ireland (*n* = 1), UK (*n* = 5) and USA (*n* = 4). Across all studies participants ranged in age from 4 to 19 years.

### Study appraisal

Study appraisal against the CASP questions is given in Tables 1 and 2.

In general studies met most criterion for quality; of 222 judgements 161 were that ‘yes’ that quality criterion is met, 41 were ‘no’ that the criterion was not met, and 20 were ‘CT’ meaning a judgement could not be conclusively made for that criterion/study. There was also observable trends across study types. Across quantitative studies for example, all were judged to have recruited in an acceptable way and almost all were judged to not have identified all important confounding factors. For qualitative studies, all were judged to have appropriately selected a qualitative approach to their research, whereas few reported or made clear that the relationship between researchers and participants had been adequately considered.

Support for the judgement presented in Tables 1 and 2 is given in Additional file 1 with some illustrative examples here.

In the study by Gordon [32] it was unclear if the study addressed a clearly focused issue and was rated as ‘can’t tell’. Two of the nine quantitative studies were judged to have identified all important confounding factors and took them into account in their design and analysis. Some caution should be attributed when using the results from Kirshnit [33], Irwin [34] and Hardy [35] as they scored ‘no’ on several quality questions.

In the qualitative studies it was unclear if the research design was appropriate for Barnett 2013 and Dismore [36]. It was also not clear if the recruitment strategies used by Azzarito [37] and Dismore [36] were appropriate. Differences in results may be explained by the differences in quality of the studies.

There is a potential bias in the quantitative studies as almost all were judged to not have identified all important confounding factors and as such analysis did not account for these. Four quantitative studies [32–35] were scored ‘no’ on the majority of the quality appraisal questions. Those studies addressed a focused issue but did not take account of confounding factors in either the design or analysis of the results and the precision of the results was questioned. Some did not list odds ratios and confidence intervals making it difficult to assess the accuracy of their findings [32–35].

Few qualitative studies reported or discussed the nature of the relationship between researchers and participants. It is therefore concerning that Barnett [38], Eime [39], Eimear-Enright [40], Fisette [41], Holt [42], Oliver [43], Stanley [44] and Totaro-Garcia [45] do not clearly provide this information. The majority (*n* = 10) of the qualitative studies scored as ‘Yes’ to sufficient rigour in data analysis indicating a well thought out and constructed process.

The estimates (barriers) reported in the two quantitative studies [30, 46], and one the qualitative study [41] which scored ‘yes’ on all CASP criteria provide high level evidence, i.e. replication of the study is unlikely to change the estimates. For qualitative studies we did not consider criterion 10 in this judgement as it is not relevant to reliability. All other studies were judged to have some factor or factors that might impact on the reliability of their estimates, i.e. further studies that do address these reliability issues may report different results.

### Barriers to sports participation

Study characteristics of the nine quantitative studies are shown in Table 3 and for the 15 qualitative studies are shown in Table 4. Eight quantitative studies focused on the generic sports [32, 33, 46–50] context with one focusing on swimming participation alone [34] in children aged 5 to 18 years. All quantitative studies made use of questionnaires and surveys. Boiche [46] investigated potential factors for dropout in sport or continuation of sport. Perry [49] sought to identify perceptions of motivators and barriers to physical activity, including sports participation. Irwin [34] also sought to identify barriers and facilitators to participation but focused on swimming. Casper [47] sought to identify constraints to participation in physical activity including sports participation and how these differ across age, gender, socioeconomic status and ethnicity. Dollman [48] focused on socioeconomic position and sport participation and how varying socioeconomic position influenced personal, social and environmental factors for participation in physical activity including sports. Gordon [32] focused on leisure activity involvement (including sports participation). All studies reported the relationship between gender and sports participation and the effect of increasing age. Two studies made use of predefined barriers for those participating in the study. One, Kirshnit [33], used electronic pagers to assist children with filling out a survey. Random messages were sent out to the pagers and on receipt children were asked to respond to a short survey. All quantitative studies made use of questionnaires and surveys. The most frequently reported barriers across the quantitative studies were ‘time’ (*n* = 4), ‘cost’ (*n* = 3), ‘opportunity/accessibility’ (*n* = 3) and ‘friends’ (*n* = 2). Where ‘friends’ was listed as a barrier studies had reported that children did not have friends to attend sessions with, or that they had no friends at the sport session and hence no one to partner with.

Across qualitative studies, one study focused on parental views of their child’s participation in sport [51] and another study looked at both parent and child perspectives of the benefits associated with participation in sport low income families [42]. Almost half of the qualitative studies focused on female experience of participation in sport only [37, 39–41, 43, 52, 53]. Azzarito [37] sought to explore the views of ethnic-minority girls and their bodies and in what space they would be physical active in their daily lives. Eime [39] made use of the socioecological model to investigate factors affecting participation in sport and physical activity in rural girls whilst Fisette [41] explored the self-identified barriers to engagement in PE and enjoyment in girls. Enright [40] also investigated the PE environment but sought to identify how a negotiated PE curriculum might look. Kimm [52] focused on a biracial cohort of sedentary females and sought to identify barriers to their participation in activity. Oliver [43] sought to understand self-identified barriers to physical activity for 5th grade females whilst Wetton [53] sought to understand the barriers to participation to develop interventions to increase team sports participation in females. Armentrout [51] sought to understand organisational barriers and personal reasons for children discontinuing participation in sport. Barnett [38] sought to understand the perception of the relationship between movement, physical activity and sport for adolescents and whether views differed according to the amount of participation. Basterfield [54] investigated how perceived barriers to participation in sports both inside and outside of school changed within the same cohort over a three year time frame. Dismore [36] investigates children’s attitudes towards PE and school sport and the influences on these for children and Holt [42] sought to understand perceptions of benefits associated with participation in youth sport in children and parents from a low income background. Quarmby [55] investigated how different family structures affected children’s time activities including sports. Stanley [44] explored lunchtime activity and children’s perceptions of factors influencing their participation. Totaro-Garcia [45] investigated the physical activity characteristics of adolescents attending a PE service. The majority of the qualitative studies reported on sport in general but one study focused on ice hockey [51]. Six studies took place in the general sports setting (can include sport both inside and outside of school and may include organised sport) [38, 39, 41, 52, 54, 55], three took place within the school setting [43–45], three took place outside of school [38, 42, 51], three focused on physical education within school [42,45,47] and two of these looked exclusively at females [37, 40]. The qualitative methods used in the study’s included interviews [36, 42, 45, 53], open survey questions [51, 52, 54], focus groups [38, 39, 44],survey and semi structured interview [55], focus group and interview [41], visual ethnography [37], participatory action research [40] and feminist action research [43]. Of the 15 qualitative studies six reported ‘time’ as a barrier to participation in sports, five reported ‘cost’, six reported ‘not being good at sport’, and six reported ‘fear of being judged/embarrassed’. Three studies reported ‘conforming to a gender stereotype’ three reported a ‘lack of resources’, and two studies reported ‘conformity’, and ‘boys dominating sports’ as barriers to participation.

The barriers identified in the quantitative studies were also identified in the qualitative studies. In contrast there were a number of barriers identified in the qualitative studies that were not identified in the quantitative studies. These additional barriers were coded as ‘politics’, ‘lack of enjoyment’, ‘self-conscious’, ‘lack of interest’, ‘competitive’, ‘conformity’, ‘bullying’, ‘lack of voice and choice’, ‘lack of motor skills’ and ‘doesn’t like strangers’, and related largely to emotion, feeling and lived experience.

**Fig. 2:**
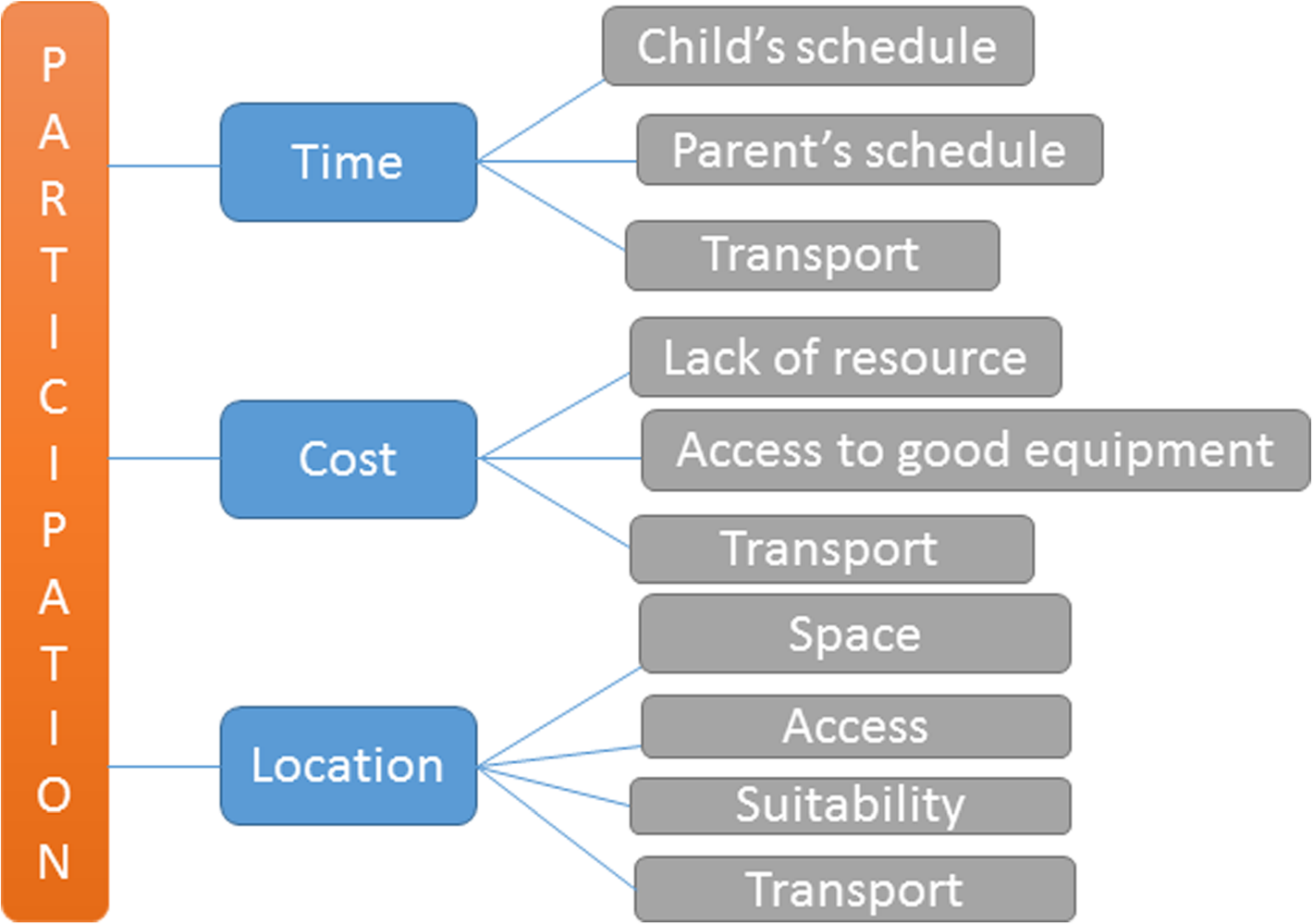
Practical barriers to participation in sport for children

Two visual representations were developed from the barriers identified across all studies included in the review. Figure 2 shows how practical barriers such as ‘transport’ and ‘location’ can link to other barriers such as ‘cost’. ‘Time’ was reported as a barrier in almost half of the studies in the review. This was either time in a child’s schedule or time for a parent to commit to taking their child to a sports session.

Figure 2 summarises the practical barriers to sports participation identified in this review. Time may be associated with time in a child’s schedule or time in a parent’s schedule. Children may wish to participate in sport but may be unable to because of a clash with their parents schedule such as working hours. The cost of participation can in some sports be fairly high and act as a barrier to some children. The cost of running a sports club may also act as a barrier to participation for children as there may be a lack of resource to facilitate provision of sporting opportunities. The type of equipment available can also influence a child’s view of participation, if it is old they are less likely to want to try a sport. Location can also influence participation, is the space suitable for the sport that is being held there or is it a compromise between cost and provision of opportunity. Transport can be linked to time, cost and location. If the location for sports participation is substantial distance from the child’s home then more time is required to travel to the venue which can incur a greater cost in transport. The most commonly reported practical barriers were used to develop the visual representation shown in Fig. 2.

**Fig. 3:**
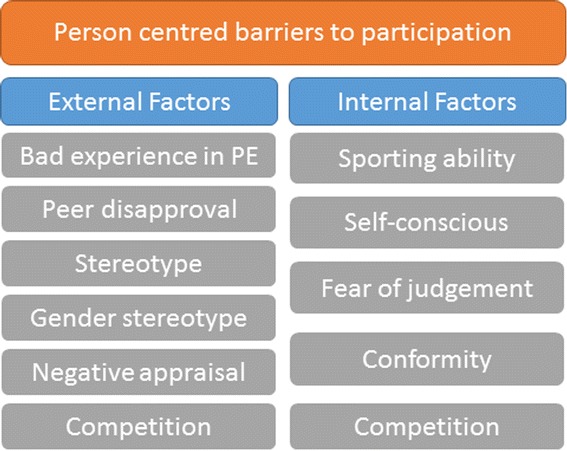
Personal barriers to children’s participation in sport

Figure 3 summarises the personal barriers to sports participation identified in this review. Person centred barriers can be linked to internal and external factors which influence them. The barriers shown in this summary were taken from the qualitative studies. The experiences of children within the studies showed that a ‘bad experience in PE, ‘ peer disapproval’, ‘stereotype, ‘gender stereotype’, and ‘negative appraisal were influenced by external factors such as peers in a class or a teacher and were grouped under ‘external factors’ in this visual representation. Reports of ‘sporting ability’, ‘self-conscious’, ‘fear of judgement’ and ‘conformity’ within the qualitative studies were linked to a child or participants reaction to a situation in the sporting context and are listed as ‘internal factors’ in this visual representation. Competition was described in the qualitative studies in different situations, there were children who were competitive (a barrier to participation for others) and there were children who were put off by competition, therefore it represents both an internal and external factor.

The barriers cost and time were frequently reported barriers in the qualitative studies. They also appeared to associate with location, transport and resources (Fig. 3); if sports facilities were located further away it was more difficult to find time for some children to participate in them. This may be due to the child’s schedule or that of their parents.

*“Well we looked at doing rock climbing out here [at the university]. [But] it’s a little bit trickier here because they have an indoor facility here but given that I [cannot drive due to medical reasons] and my wife’s in school so she’s not really there to drive us, getting out there and back on the bus would shoot three hours. Right? Like here it’s a good 40 minutes one way, then an hour lesson and then 40 minutes back.” – Holt* et al. [42].

Traveling further can cost more in terms of fuel or public transport, for example a parent in the study by Armentout et al. [51] stated, *“He didn’t want to quit playing, I told him we can’t afford it and we didn’t have the time”*, when talking about ice hockey practice.

A parent in the study by Holt et al. [42] reported on the additive cost of travel, training costs, and costs of competitions as a barrier:

*“Yeah. And each time she [daughter] does it, there’s a cost…its $5 for every race or is it even more. And I drive her there [another cost]. I don’t think that [the organizers] realize how sometimes those costs actually prevent my kids from joining running club... You don’t want your child to be labelled necessarily as the one that can’t afford it.” – Holt* et al. [42].

Higher cost was associated with particular sports in some of the studies, for example ice hockey was described as expensive [42, 51].

*“cost to play sport [ice hockey] unreasonable” – Armentrout et all* [41].

*“Like we’re not even talking hockey [an expensive sport].” – Holt* et al. [42].

*“It was an expensive program for the amount of practice and ice time available*” -.

*Armentrout* et al. [51].

Single parents reported cost as a barrier to participation in sport for their child. This was also reported by parents in a partnership but was seen as a greater barrier by single parents.

*“When [name of children] grow older the price changes sometimes. And the fees get more costly. And we went through a separation, me and my husband, and then you know sometimes it gets difficult. But I don’t want [my son] to know [my financial circumstances]. I would work extra hard for him to pay for his sports.”- Holt* et al. [42].

Time was frequently reported as a barrier in different contexts. Armentrout et al. [51] reported a lot of feedback on amount of time for ice hockey practice.

*“Early morning practices were tough”, “too much practicing for young kids”, “unreasonable practice times” and “too much time commitment”.*

Other parents reported their job commitments as affecting participation for their children.

*“… our son has to adjust to our schedule unfortunately because we can’t change it and I can’t help it. I have three jobs at the same time because we have to pay our bills and I have to support my family here.” – Holt* et al. [42].

Time was a barrier that related to the status of the parent; whether they were in a partnership or were a single parent had an impact on their child’s sports participation. Children with a single working parent found their parents schedule a barrier to their participation in sport.

*“My mum’s job [referring to barriers to activity], she gets back at like half five so if I want to go anywhere to do anything it’s normally too late so instead like I just play X box and stay in when she’s not back.” – Quarmby* et al. [55].

The practical barriers (Fig. 2) may be more easily overcome than the personal barriers (Fig. 3). Practical barriers are potentially linked to more changeable situations such as time a child has to spend on the sport; this can change at various times during their school and college lives. Location can also be adaptable depending on the type of sport the child wishes to engage in.

Some of the personal barriers identified include ‘conformity’, ‘fear of judgement’, ‘disapproval’ and ‘gender stereotypes’. ‘Conforming to gender stereotypes’ was a barrier reported in three of the papers included in the review [41, 43, 53]. ‘Conforming to parent’s expectations’ and ‘media expectations in terms of gender stereotype’ were mentioned in two studies.

*“Well yeah, it is for girls, but it’s just in their [my parents’] mind; it isn’t…I don’t think any girls would go out and play with a football, because it’s not like what it is supposed to be seen as, people playing football.” – Azzarito* et al. [37].

“*Sport is seen as a manly thing to do… they [media] don’t see it as a girly thing”.*

*– Wetton* et al. [53].

## Discussion

The nine quantitative studies included in the review used questionnaires to collect information on the barriers to children’s participation in sport. Of those, only Casper [47] and Dollman [48] scored ‘yes’ to all questions on the quality appraisal. All studies addressed a clearly focused issue, with the possible exception of Gordon [32] where the study aimed to answer numerous research questions. The quantitative studies generally predefined a set of barriers for the participants to choose from or rate. This may not provide a comprehensive picture of the barriers to participation in sport for children. A qualitative approach where the participant can share and discuss their own personal thoughts and experiences as to the barriers to participation in sport further represent the barriers faced. The results from the quantitative studies are likely to be biased towards the barriers identified by the researchers for these studies. The barriers emerging from the quantitative studies relate more to the practical barriers such as lack of time and high cost as opposed to the personal or psychosocial barriers faced by children. Lack of parental support was identified as a barrier to participation in sport for children; here and in a previous review on motivations to take part in sport [56]. A study in 8th grade children in Chile identified children with a more sedentary lifestyle were more likely to have parents who did not support them to play sport or to be physically active [3]. A study in the USA of children aged 10 to 14 years identified the mother of the children as an influential figure in whether they participated in sport, be this as a role model or as support for starting a new program of exercise [57]. Had a qualitative approach been used more information could have been gathered on exactly how parents influence children’s participation levels.

Armentrout [51], Kimm [52] and Basterfield [54] used a survey / questionnaire approach where the questions were open ended. Armentrout [51] conducted a study involving parents of children participating in ice hockey in the USA. This study related to a specific sport rather than a general sport and as such the findings may be difficult to apply to other sports. Some general barriers such as ‘cost’, ‘lack of enjoyment’ and ‘location’ were identified which are not sport specific but barriers such as ‘availability of the ice rink’ would not be relevant to all sports [51]. Kimm [52] also used the survey approach in a general sports context but the study focused on females. The barriers identified by males and females are likely to be different. Males are reportedly more active than females at all ages from youth to adolescence [18, 20, 58]. The study by Kimm [52] was conducted in the USA and barriers such as ‘embarrassment’ and ‘no one to go with’ were identified. These barriers were also identified in other studies in the review involving both males and females so these are not gender specific but may affect one gender more than the other. Basterfield [54] also identified ‘Being shy’ and ‘fear of making a mistake’ as barriers to participation and this study involved both male and female participants. Armentrout [51] and Kimm [52] made use of surveys and the relationship between researcher and participant in the quality appraisal was scored as ‘no’, this is unlikely to affect the quality of these studies given the surveys would have been completed by the participants independent of the researcher. Although the findings from both studies may be difficult to apply outside of their research context as one study is sport specific and the other is gender specific. It may have been worthwhile to have a different sport for comparison with ice hockey [51] and to have a sample of males for comparison [52].

Barnett [38] made use of focus groups with 16 to 18 years olds in Australia to explore sport in schools. It was judged unclear whether the research design was appropriate to address the aims of the research and whether the data were collected in a way that addressed the research question. Given the use of focus groups consideration of the relationship between the researchers and the participants should be reported, however briefly. That said ‘not being good at sport’ was identified as a barrier in this study [38] and ‘perceived competence’ was identified as a barrier in another [44]. Stanley [44] also made use of focus groups with children aged 10 to 13 years in Australia to discuss sports participation in general. The relationship between researcher and participants was not reported. Study quality did not appear to influence the results reported. For example Barnett [38] and Stanley [44] identified some similar barriers to other studies in the review. Fisette [41] also made use of focus groups but combined them with interview data. Again this study had not accounted for researcher influence on the focus groups [41].

Gender stereotype was identified as a barrier to participation. This was reported more frequently by females [41, 53]. Girls were less likely to participate in sport if boys were present as they felts “girls are supposed to do girly things” [41]. If they did participate there was a sense of having to prove themselves to the boys to be able to participate. There were also issues of males dominating the spaces used for sport within school and females not being “allowed” to engage or participate [43]. Oliver [59] made use of a feminist active research approach finding that only females felt isolated and as though they lacked a voice in the school sporting context, so lost interest in participation [40]. Enright [40] made use of participatory action research involving females aged 14 to 19 focusing on the school physical education environment. Existing stereotypes can be very difficult for females in sport to overcome [53, 60]. As such it is important to demonstrate positive female role models within the sporting context that can inspire both girls and boys [57, 60].

Lack of time, high cost and location were common barriers. Children from poorer backgrounds and those from single parent families are more likely to be affected by these barriers [18, 19, 42, 44, 45]. Particular sports can be expensive to participate in given the need for specialist equipment and location (e.g. ice hockey) and practice time isn’t always enough to keep the child engaged in the sport or to give them a sense of achievement [51]. Children may also have to choose between sports as there may not be enough time for them to do all sports they would like to do, especially considering transport time and practice time parents may not be able to accommodate [42, 53].

The major barriers identified in this review in both qualitative and quantitative studies were ‘time’ and ‘cost’ which have already been discussed. The barriers identified in addition to these in the qualitative studies were ‘not being good at sport’, ‘fear of being judged/embarrassed’ and ‘conforming to a gender stereotype’. Children who were not good at sport (or who felt they were not good at sport) were less likely to participate [38, 53]. This has been identified as a barrier to participation in physical activity in other studies [56]. ‘Fear of judgement’ or ‘gender stereotype’ has also been identified in other studies. Evidence shows that girls are less physically active than boys regardless of age [18, 19, 58]. The reasons for this discrepancy have been linked to gender stereotypes [43, 61].

There are a number of common limitations in the literature. For example, only two of the quantitative studies were judged to have considered all confounding factors [47, 48]. Of the quantitative studies, four were judged during appraisal not to be applicable to the local population [32–35]. The literature reviewed here compares findings from very heterogeneous settings, where the specific barriers might be very different ones. For example, the barrier most often mentioned was ‘lack of time’. However, lack of time implies that other activities, be it schoolwork, computer games, household chores, or time with family are given higher priority than sport by the child or parent; theses example would require quite different facilitators to overcome. To account for this in future survey studies should captured ‘time’ according to the specific meaning be it leisure time, school activity, etc. A few of the quantitative studies scored a ‘No’ on quality appraisal for precision of the results as not all important information such as confidence intervals, odds ratios and standard deviations were reported. Items required by the CASP appraisal system. A significant portion of the qualitative studies scored a ‘No’ on the CASP appraisal for taking account of the relationship between researcher and participants [38, 40, 44, 45, 51–53, 55, 62, 63] whilst two were judged as cannot tell [36, 41]. To score a ‘No’ the study failed to report critical evaluation of the researcher of their role, how questions were formed, how data was sampled and collected, location choice and how the researcher responded to events during the study and if they considered the impact of changing parts of the research design. The researchers own lived experiences shapes their epistemology and the way in which they approach the research. As such the researcher brings certain preconceived ideas to a study. For those not explaining or accounting for this it is unclear the impact the researcher has had on the results of the study. Evidence shows that researchers can influence their participants and that good qualitative research happens when the researcher is reflexive and truly understands their impact on the study and analysis [64–66]. In a survey for example the researcher has less influence on the answers provided than they might have in a one-to-one interview or focus groups the researcher can influence the outcome of the process. Future studies should ensure they include a section on reflexivity in their methods to ensure there is transparency in their methodology and approach. Reflecting on quality appraisal tools such as CASP will help in project design to circumvent later criticism against such standards. However the CASP tools were not used within this review to include or exclude studies but were used to provide a guide as to the quality of the studies included in the review.

This review has shown that studies specifically investigating barriers to participation in sport, as opposed to broader constructs such as physical activity, are few and there are a number of unanswered questions. It is unclear for example how participation in PE influences sports participation later in life. Study’s investigating the link between physical education and sports participation outside of school were lacking from the studies identified as eligible for this review. Studies evaluating barriers to participation in PE have more commonly focussed on those faced by young females, and little is known of barriers to participation from the young male perspective. Again, there is an indication for more balance in the literature.

Given the importance of physical activity and what sport can contribute to this it is essential to address the barriers in this review. There are a number or practical and personal barriers affecting children’s participation in sport. It may be easier to address practical barriers to drive up participation in sport for children than the personal barriers. For example transport, location and time were mentioned frequently. These barriers might be addressed in some cases by making (more) local sports sessions available to children at times such as immediately after school or during the school day. There cannot of course be any ‘one-size--fits-all’ policy for overcoming barriers to sports participation. We can only therefore conclude the need to think at a local level how the barriers identified in this review affect children sports participation and how these specific barriers might be affected by changes locally, whilst being mindful of the potential impact of national and international initiatives.

Some recent initiatives such as the introduction of the PE and sport premium for primary schools in the UK to provides funding for sport participation for primary school children may reduce barriers for some children [67]. The impact of these initiatives will have to be carefully monitored. The UK Government has also introduced a new strategy for an Active Nation to help combat the rising levels of inactivity and increase levels of sport participation in the UK. Other international initiatives are also attempting to increase participation levels in sport and physical activity. The European Union has a dedicated week of sport to promote physical activity and sport participation across Europe, this is alongside the European union policy on sport which includes a section on health and participation in sport [68]. The Australian Government released a strategic plan for sport in 2011 for which the primary goal was to increase participation in sport across Australia and to increase participation in under-represented groups [69].

Future research and policy should focus on more detailed information about ‘Why children do not participate in sport’ and ‘why they discontinue participation’. This is essential to developing interventions to improve sports participation for deaf and hard of hearing children, and ultimately thereby their health and development.

## Conclusions

Policy makers, parents and teachers should all be aware that ‘cost’ and ‘time’ are key barriers to participation in sport. More local sports opportunities are needed where costs are reduced. Schools and local clubs could better work together to provide more affordable local opportunities to increase children’s participation in sport.

In conclusion, this systematic review identifies time, cost, and location as prominent practical barriers to children’s participation in sports. Policy makes should be aware of this when planning provisions. Person-centred barriers such as peer disapproval and stereotyping also significantly affect participation. These require a cultural change in the sports environment through training and support to those delivering sports sessions.

### Additional file

Additional file 1: Table S1. Quality appraisal of quantitative and qualitative studies using the CASP tool. Contains information on the quantitative studies included in the review and the quality appraisal of these. Table 2 contains the quality appraisal information for the qualitative studies. (XLSX 26 kb)

## Appendix

Appendix Example electronic search conducted in EMBASE, June 2015.

1. barrier*.mp.
2. stop*.mp.
3. prevent*.mp.
4. 1 or 2 or 3
5. participat*.mp.
6. taking part.mp.
7. 5 or 6
8. sport/ or sport*.mp.
9. PE.mp.
10. child*.mp.
11. adolescen*.mp.
12. young person*.mp.
13. 10 or 11 or 12
14. "physical education".mp. or physical education/
15. 8 or 9 or 14
16. 4 and 7 and 13 and 15

## Abbreviations

CASP: Critical Appraisal Skills Programme
PA: Physical Activity
PE: Physical Education
UK: United Kingdom
